# A web-based calculator for predicting the prognosis of patients with sarcoma on the basis of antioxidant gene signatures

**DOI:** 10.18632/aging.203885

**Published:** 2022-02-10

**Authors:** Kun Quan, Zhiyou Cao, Qiang Xu, Meisong Zhu, Xuqiang Liu, Min Dai

**Affiliations:** 1 Department of Orthopedics, The First Affiliated Hospital of Nanchang University, Artificial Joints Engineering and Technology Research Center of Jiangxi Province, Nanchang, Jiangxi, China

**Keywords:** sarcoma, prognostic model, overall survival, disease-free survival, antioxidant genes

## Abstract

Background: Oxidative stress plays a critical role in tumorigenesis, tumor development, and resistance to therapy. A systematic analysis of the interactions between antioxidant gene expression and the prognosis of patients with sarcoma is lacking but urgently needed.

Methods: Gene expression and clinical data of patients with sarcoma were derived from The Cancer Genome Atlas Sarcoma (training cohort) and Gene Expression Omnibus (validation cohorts) databases. Least absolute shrinkage, selection operator regression, and Cox regression were used to develop prognostic signatures for overall survival (OS) and disease-free survival (DFS). Based on the signatures and clinical features, two nomograms for predicting 2-, 4-, and 6-year OS and DFS were established.

Results: On the basis of the training cohort, we identified five-gene (*CHAC2*, *GPX5*, *GSTK1*, *PXDN*, and *S100A9*) and six-gene (*GGTLC2*, *GLO1*, *GPX7*, *GSTK1*, *GSTM5*, and *IPCEF1*) signatures for predicting OS and DFS, respectively, in patients with sarcoma. Kaplan–Meier survival analysis of the training and validation cohorts revealed that patients in the high-risk group had a significantly poorer prognosis than those in the low-risk group. On the basis of the signatures and other independent risk factors, we established two models for predicting OS and DFS that showed excellent calibration and discrimination. For the convenience of clinical application, we built web-based calculators (OS: https://quankun.shinyapps.io/sarcOS/; DFS: https://quankun.shinyapps.io/sarcDFS/).

Conclusions: The antioxidant gene signature models established in this study can be novel prognostic predictors for sarcoma.

## INTRODUCTION

Sarcomas are a diverse group of rare and heterogeneous malignancies of mesenchymal origin [1, 2]. They comprise 50 histological types that can be broadly classified into bone sarcomas, including osteosarcoma, Ewing’s sarcoma, chondrosarcoma, and soft-tissue sarcomas, including liposarcoma, leiomyosarcoma, undifferentiated soft-tissue sarcoma, fibrosarcoma, and synovial sarcoma [3, 4]. In the USA, 13,460 new soft-tissue sarcoma and 3,610 new bone sarcoma cases are reported annually [5]. In recent decades, great efforts have been made in sarcoma research. However, there have been no significant improvements in sarcoma treatment for nearly 30 years. In addition, the prognosis of sarcoma is not satisfactory due to local recurrence and distant metastases [6]. Therefore, it is imperative to develop reliable prognostic tools to accurately estimate patient outcomes and improve personalized therapy for patients with sarcoma.

Oxidative stress is the result of excess reactive oxygen species (ROS) due to an imbalance between ROS production and antioxidant responses [7, 8]. ROS are implicated in tumor growth, invasion, and metastasis, and cellular resistance to therapy [9]. ROS are produced inside the cells from both endogenous and exogenous sources [10]. Although low levels of ROS can be beneficial, excessive accumulation can promote cancer [11]. It is becoming increasingly evident that ROS play crucial roles in the survival, proliferation, invasion, angiogenesis, and metastasis of tumor cells [12–14]. Under normal conditions, ROS production is balanced by ROS scavenging through the antioxidant defense machinery, resulting in redox homeostasis. Studies using preclinical and clinical models have indicated that antioxidants reduce cancer risk [15, 16]. Antioxidant-related genes may be related to tumorigenesis as they play a major role in regulating oxidative stress and protecting against ROS [17]. Studying the relationship between antioxidant genes and tumorigenesis may facilitate the discovery of novel targets for predicated prognosis and treatment [18]. However, the prognostic values of antioxidant-related genes and their biological functions in sarcomas remain rudimentary and inconclusive.

In the present study, we aimed to construct and validate prognostic antioxidant-related gene signatures for overall survival (OS) and disease-free survival (DFS) in sarcoma patients using gene expression profiling data from The Cancer Genome Atlas (TCGA) and Gene Expression Omnibus (GEO) databases. In addition, we performed functional analysis and compared tumor immunity between high- and low-risk groups. On the basis of the signatures and other risk factors, we constructed predictive nomogram models. For convenient clinical application, we built web-based calculators.

## RESULTS

### Establishment of prognostic signatures based on antioxidant genes

**Figure 1:**
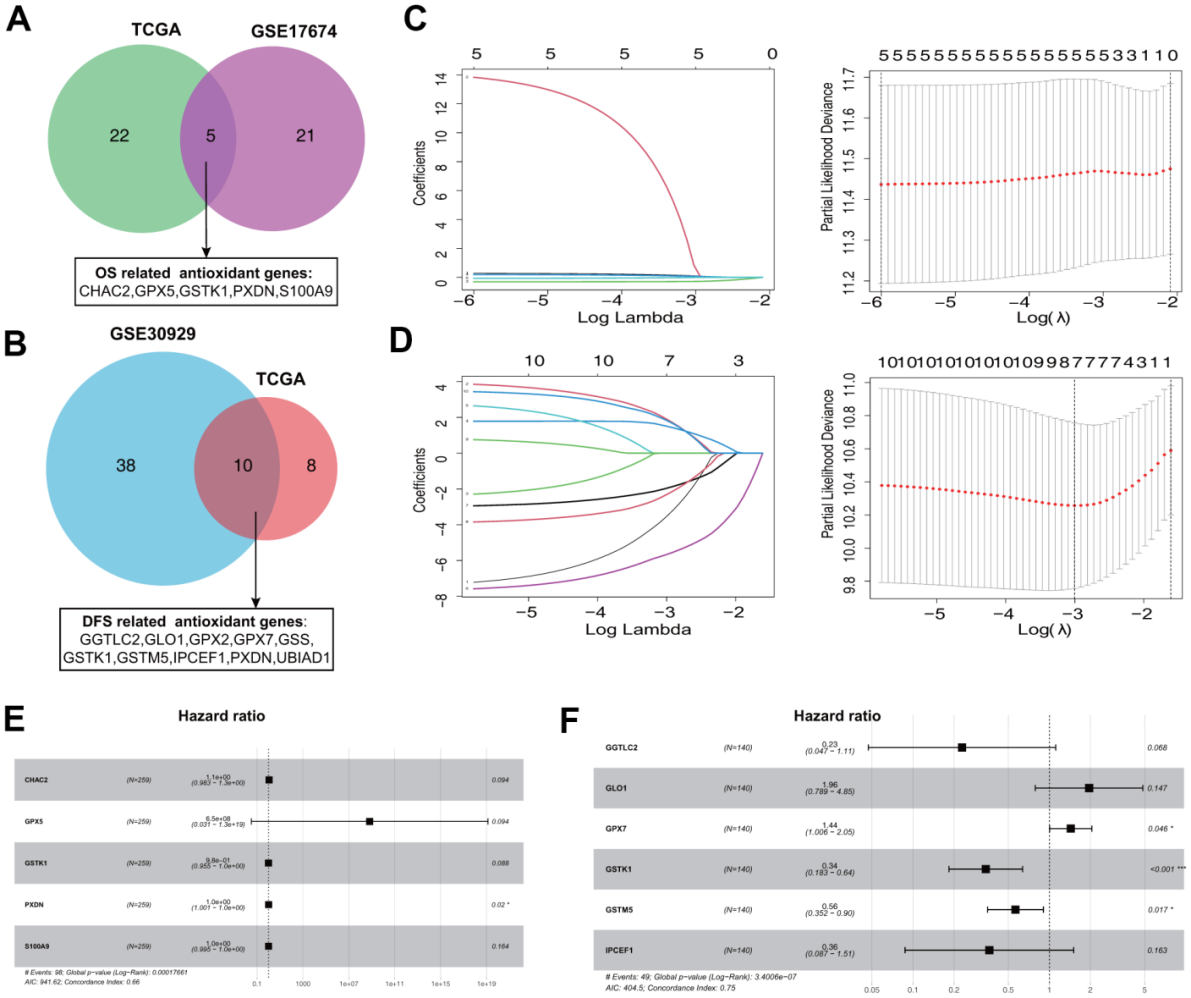
**Establishment of prognostic antioxidant gene signatures in the training cohort.** (**A**) Overlapping overall survival (OS)-related antioxidant genes in The Cancer Genome Atlas (TCGA) Sarcoma (TCGA-SARC) and Gene Expression Omnibus (GEO) GSE17674 datasets. (**B**) Overlapping disease free survival (DFS)-related antioxidant genes in the TCGA-SARC and GSE30929 datasets. (**C**) Least absolute shrinkage and selection operator (LASSO) regression analysis to screen the antioxidant genes for the predictive OS signature. (**D**) LASSO regression analysis to screen the antioxidant genes for the predictive DFS signature. (**E**) Forest plot of multivariate Cox regression analysis of the genes in the OS signature. (**F**) Forest plot of multivariate Cox regression analysis of the genes in the DFS signature.

To narrow down candidate antioxidant genes, we performed univariate Cox regression analysis and identified 27 and 26 OS-related antioxidant genes in the TCGA-Sarcoma (TGCA-SARC) and GSE17674 datasets, respectively (Supplementary Table 2). Among these, five overlapping genes were selected for establishing the OS signature (Figure 1A). Similarly, we identified 18 and 48 DFS-related antioxidant genes in the TCGA-SARC and GSE30929 datasets (Supplementary Table 3), respectively; 10 overlapping genes were selected for establishing the DFS-related signature (Figure 1B).

The five OS-related and 10 DFS-related antioxidant genes were subjected to tenfold cross-validated LASSO regression to generate the best gene model. The LASSO coefficients were plotted against the log(k) values, and five OS-related genes and seven DFS-related genes were selected (Figure 1C, 1D). On the basis of multivariate Cox regression analysis results, five genes (*CHAC2*, *GPX5*, *GSTK1*, *PXDN*, and *S100A9*) associated with OS and six genes (*GGTLC2*, *GLO1*, *GPX7*, *GSTK1*, *GSTM5*, and *IPCEF1*) associated with DFS were selected for establishing risk signatures (Figure 1E, 1F). The prognostic risk score formula was specifically constructed by the multivariate Cox regression analyses (Tables 1, 2).

**Table 1:** Overall survival-related antioxidant gene signature identified through LASSO and Cox regression.

**Gene symbol**	**Coefficient**	**Gene product**
*CHAC2*	0.103700036	ChaC glutathione-specific gamma-glutamylcyclotransferase 2
*GPX5*	20.29092431	glutathione peroxidase 5
*GSTK1*	–0.021559973	glutathione S-transferase kappa 1
*PXDN*	0.00814439	peroxidasin
*S100A9*	–0.002047307	S100 calcium-binding protein A9

**Table 2:** Disease-free survival-related antioxidant gene signature identified through LASSO and Cox regression.

**Gene symbol**	**Coefficient**	**Gene product**
*GGTLC2*	–1.474979614	gamma-glutamyltransferase light chain 2
*GLO1*	0.671088392	glyoxalase I
*GPX7*	0.361529489	glutathione peroxidase 7
*GSTK1*	–1.073983673	glutathione S-transferase kappa 1
*GSTM5*	–0.572788673	glutathione S-transferase mu 5
*IPCEF1*	–1.013381673	interaction protein for cytohesin exchange factors 1

### Evaluation of the signatures in the training and validation cohorts

Based on the established OS and DFS risk signatures, the risk score for each patient with sarcoma was calculated. The median score was set as the cutoff value for categorizing sarcoma patients into low- (≤ median score) and high-risk (> median score) groups.

**Figure 2:**
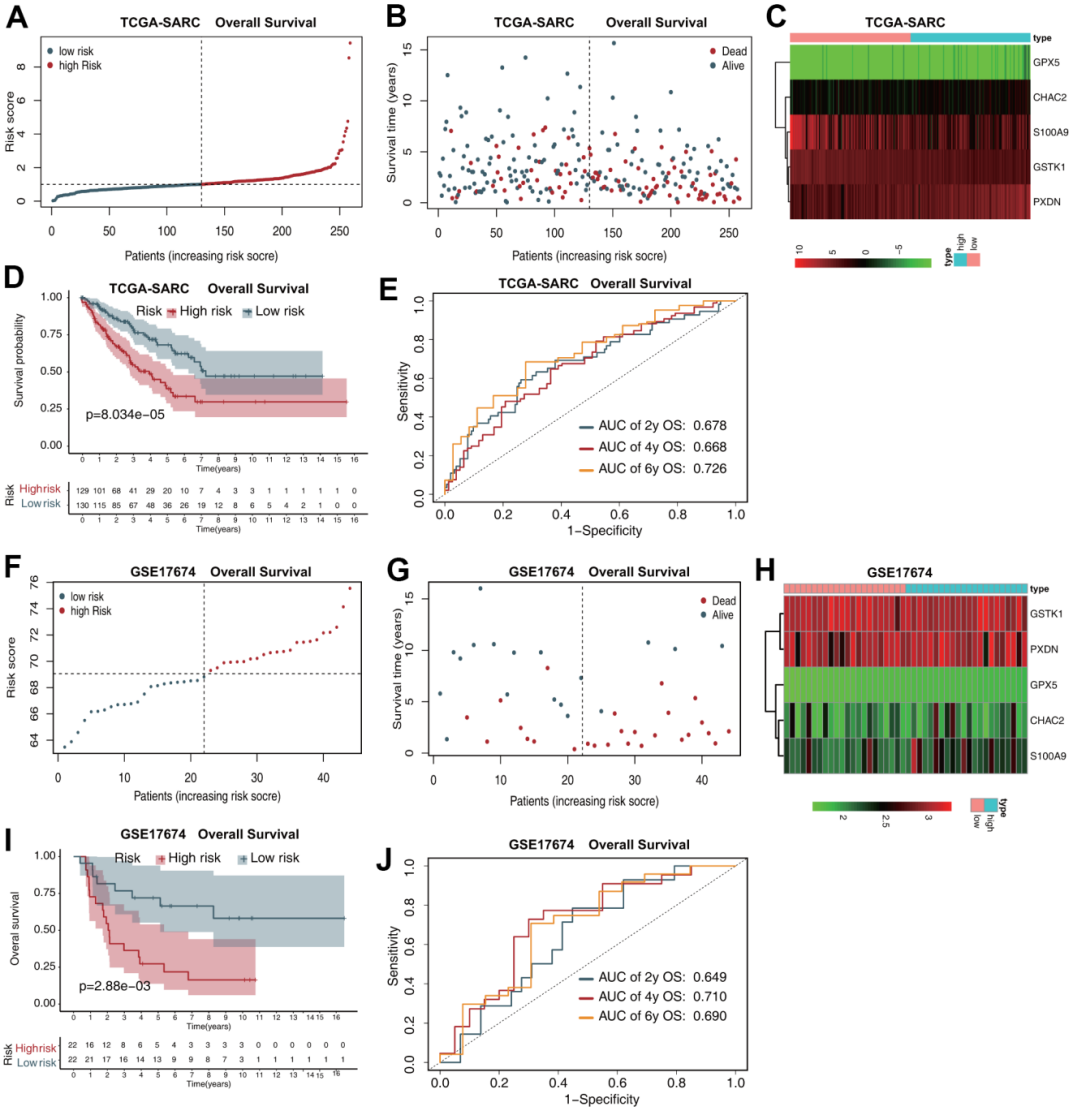
**The five-gene prognostic signature predicts the OS of patients with sarcoma.** (**A**) Risk score distribution in the TCGA-SARC dataset. (**B**) OS time distribution in the TCGA-SARC dataset. (**C**) Expression heatmap of the five genes in the TCGA-SARC dataset. (**D**) Kaplan–Meier analysis of OS based on the signature in the TCGA-SARC cohort. (**E**) Receiver operating characteristic (ROC) analysis of OS prediction in the TCGA-SARC cohort. (**F**) Risk score distribution in the GSE17674 dataset. (**G**) OS time distribution in the GSE17674 dataset. (**H**) Expression heatmap of the five genes in the TCGA-SARC dataset. (**I**) Kaplan–Meier analysis of OS based on the signature in the GSE17674 cohort. (**J**) ROC analysis of OS prediction in the GSE17674 cohort.

The risk score distribution for OS prediction in the TCGA-SARC dataset is shown in Figure 2A, and the distribution of OS status ranked by risk score is presented in Figure 2B. The expression profiles of the five antioxidant genes in the two groups are shown in Figure 2C. Survival analysis revealed that patients in the low-risk group had a better OS than those in the high-risk group (Figure 2D). In time-dependent receiver operating characteristic (ROC) analysis, the areas under the ROC curves (AUCs) for 2-, 4-, and 6-year OS were 0.678, 0.668, and 0.726, respectively (Figure 2E).

The risk score distribution for OS prediction in the GSE17674 dataset is shown in Figure 2F, and the distribution of OS status ranked by risk score is presented in Figure 2G. The expression profiles of the five antioxidant genes in the two groups are shown in Figure 2H. Survival analysis revealed that patients in the low-risk group had a better OS than those in the high-risk group (Figure 2I). The AUCs in time-dependent ROC analysis for 2-, 4-, and 6-year OS were 0.649, 0.710, and 0.690, respectively (Figure 2J).

**Figure 3:**
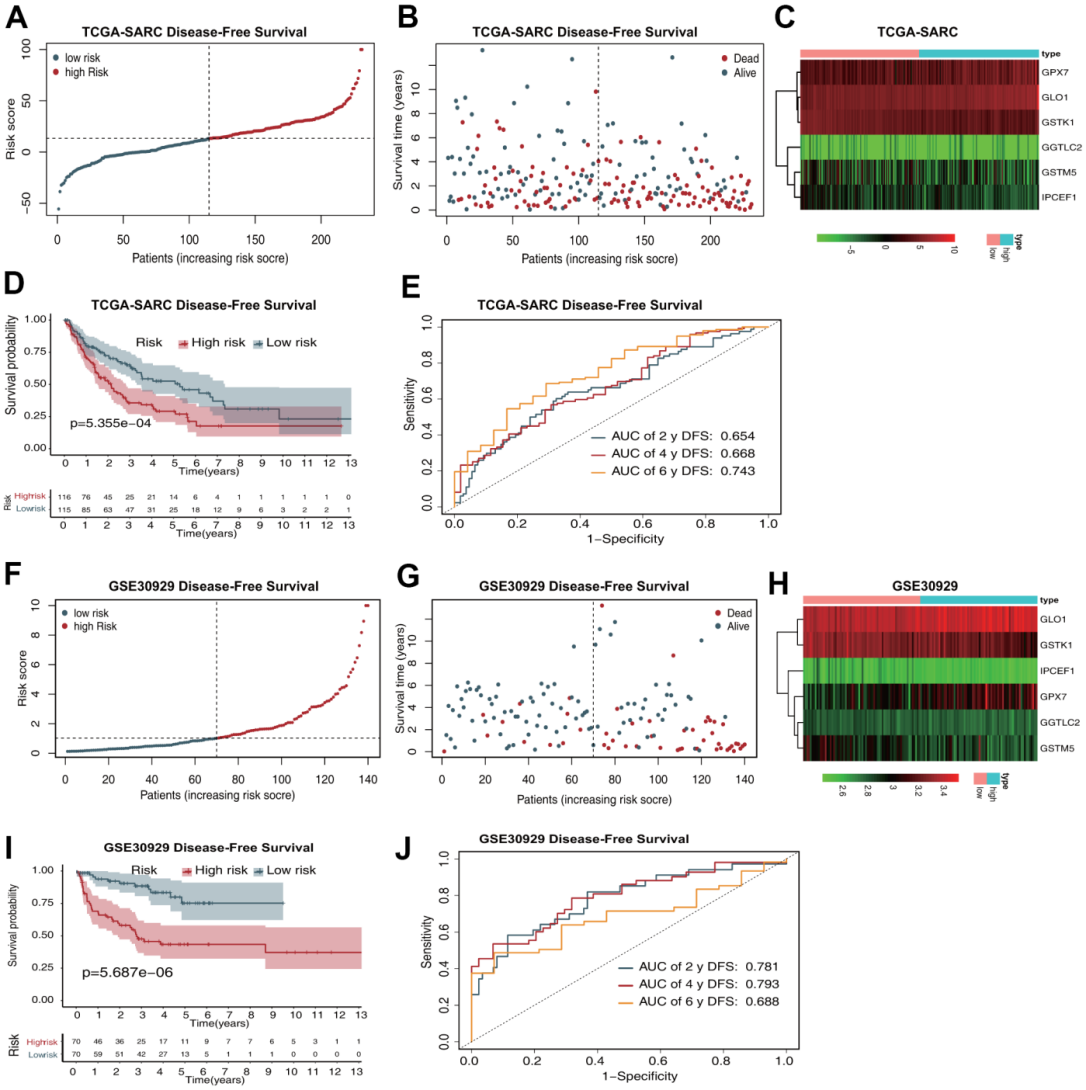
**The six-gene prognostic signature predicts the DFS of patients with sarcoma.** (**A**) Risk score distribution in the TCGA-SARC dataset. (**B**) OS time distribution in the TCGA-SARC dataset. (**C**) Expression heatmap of the six genes in the TCGA-SARC dataset. (**D**) Kaplan–Meier analysis of OS based on the signature in the TCGA-SARC cohort. (**E**) ROC analysis of OS prediction in the TCGA-SARC cohort. (**F**) Risk score distribution in the GSE30929 dataset. (**G**) OS time distribution in the GSE30929 dataset. (**H**) Expression heatmap of the six genes in the TCGA-SARC dataset. (**I**) Kaplan–Meier analysis of OS based on the signature in the GSE30929 cohort. (**J**) ROC analysis of OS prediction in the GSE30929 cohort.

The risk score distribution for DFS prediction in the TCGA-SARC dataset is shown in Figure 3A, and the distribution of DFS status ranked by risk score is presented in Figure 3B. The expression profiles of the five antioxidant genes in the two groups are shown in Figure 3C. Survival analysis revealed that patients in the low-risk group had a better DFS than those in the high-risk group (Figure 3D). The AUCs in time-dependent ROC analysis for 2-, 4-, and 6-year DFS were 0.654, 0.668, and 0.743, respectively (Figure 3E).

The risk score distribution for DFS prediction in the GSE17674 dataset is shown in Figure 3F, and the distribution of DFS status ranked by risk score is presented in Figure 3G. The expression profiles of the five antioxidant genes in the two groups are shown in Figure 3H. Survival analysis revealed that patients in the low-risk group had a better DFS than those in the high-risk group (Figure 3I). The AUCs in time-dependent ROC analysis for 2-, 4-, and 6-year DFS were 0.781, 0.793, and 0.688, respectively (Figure 3J).

### Gene mutation analysis and gene set enrichment analysis (GSEA)

**Figure 4:**
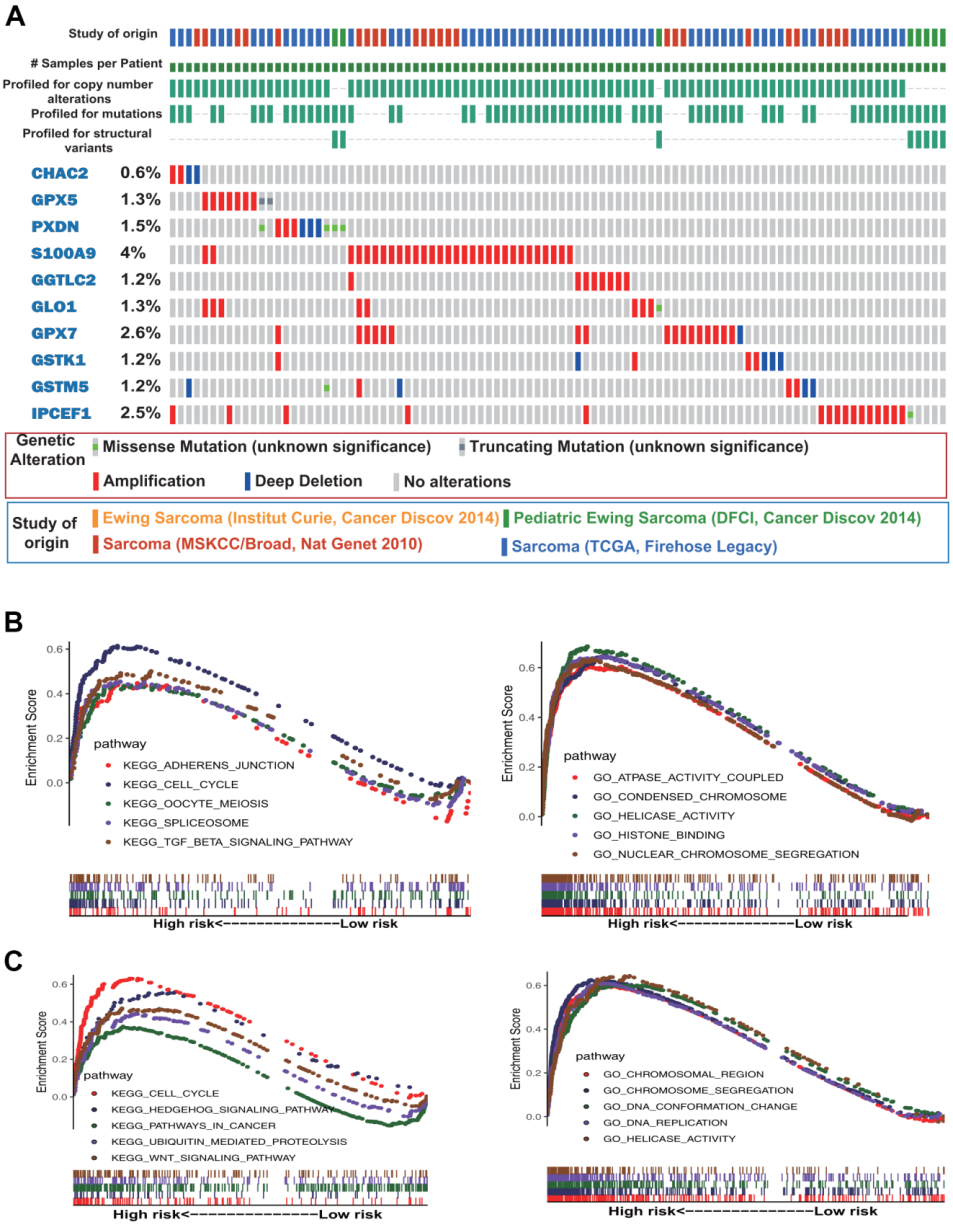
**Gene mutation and gene set enrichment analyses.** (**A**) Mutational profiles of the antioxidant genes included in the signature of sarcoma (obtained from cBioPortal). (**B**) Gene ontology (GO) functional annotation terms and Kyoto Encyclopedia of Genes and Genomes (KEGG) pathways enriched in the OS high-risk group. (**C**) GO functional annotation terms and KEGG pathways enriched in the DFS high-risk group.

Gene mutation analysis of the 10 antioxidant genes included in the OS and DFS signatures showed that *S100A9*, *GPX7*, *IPCEF1*, *PXDN*, and *GPX5* were the most frequently mutated genes. Notably, amplification was the most common type of mutation, and *S100A9*, *IPCEF1*, and *GPX7* were frequently amplified in sarcoma (Figure 4A). Subsequently, we performed GSEA based on Gene Ontology (GO) functional annotation and Kyoto Encyclopedia of Genes and Genomes (KEGG) pathway annotation in the low- and high-risk groups. The top five GO terms and KEGG pathways that were significantly enriched in the high-risk group are shown in Figure 4B, 4C.

### Immune characteristics of sarcomas

**Figure 5:**
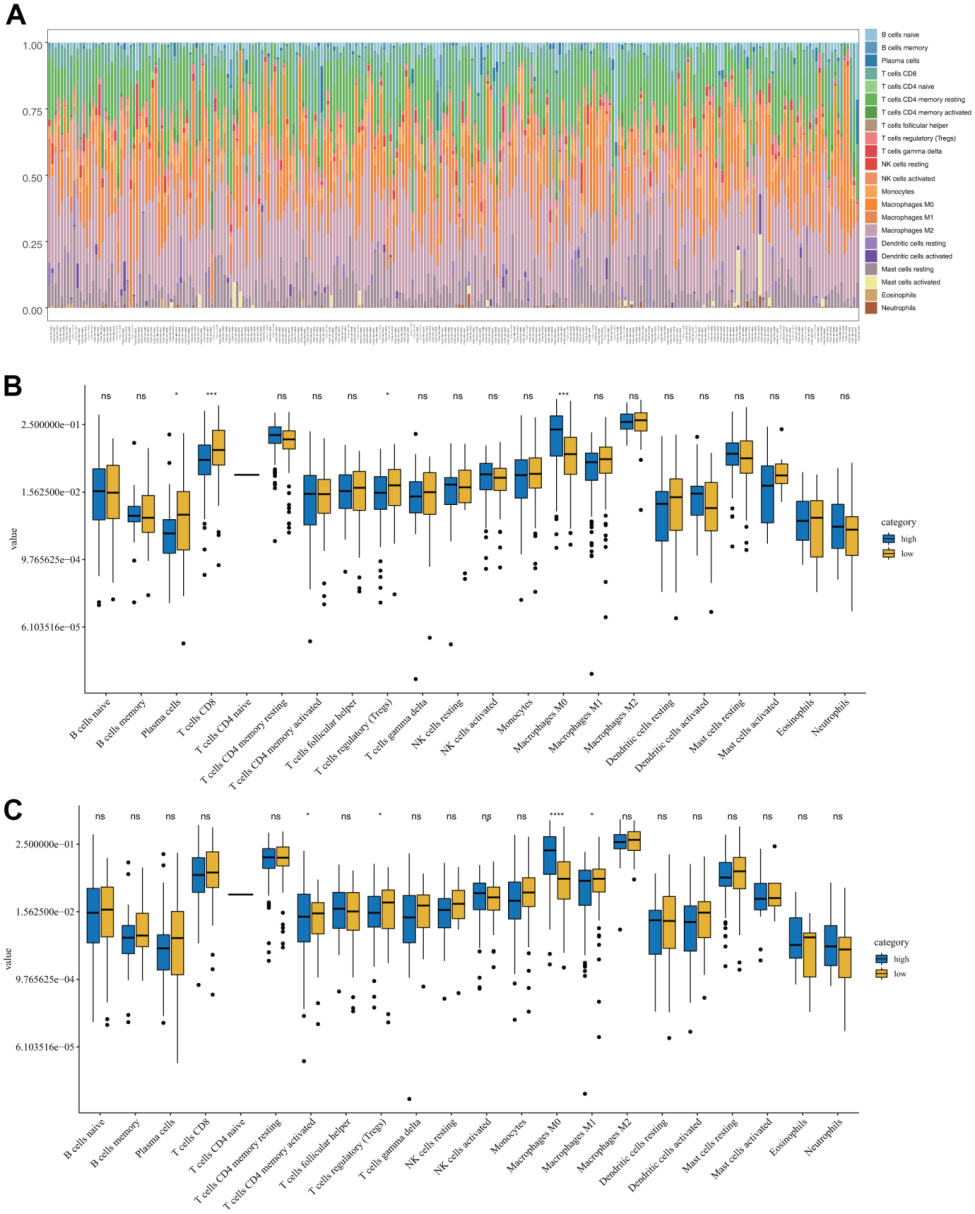
**Immune characteristics of sarcomas in the low- and high-risk groups.** (**A**) Tumor-infiltrating immune cell composition in each sarcoma sample. (**B**) Distribution of 22 types of immune cells in the OS low- and high-risk groups. (**C**) Distribution of 22 types of immune cells in the DFS low- and high-risk groups.

CIBERSORT was used to compare the level of infiltration of 22 immune cell types between the low- and high-risk groups in the TCGA-SARC samples. Figure 5A shows the tumor-infiltrating immune cell composition in each sample. Among the 22 immune cell types, the levels of infiltration of plasma cells, non-activated macrophages (M0), and cytotoxic CD8+ T cells were significantly different between the OS low- and high-risk groups (Figure 5B). For DFS, the levels of infiltration of memory CD4+ T cells and M0 and proinflammatory (M1) macrophages were significantly different between the two groups (Figure 5C).

### Development of individualized prediction nomograms and web-based calculators

To construct the nomograms, independent OS and DFS prognostic factors were identified by Cox proportional hazards regression analyses and further analyzed using multivariate Cox regression. Based on the results, age, metastasis, margin status, multifocal indicator, and antioxidant gene signature were independent predictors of OS (Table 3), whereas metastasis, margin status, and antioxidant gene signature were independent predictors of DFS (Table 4). These factors were integrated into the nomogram.

**Table 3:** Overall survival based on the antioxidant gene signature and clinical data for sarcoma.

	**Univariate Cox analysis**				**Multivariate Cox analysis**		
	**HR**	**95%CI**	***P***		**HR**	**95%CI**	***P***
**Age**	1.020	1.005–1.036	**0.010**		1.040	1.017–1.062	**0.000**
**Sex** (Female)	0.853	0.571–1.275	0.439				
**Ethnicity** (Asian)							
Black	1.085	0.132–8.924	0.939				
White	0.791	0.108–5.771	0.817				
**Histological type** (DLP)							
LMS	0.844	.513–1.389	0.505				
MFS	0.703	.328–1.507	0.365				
Other	0.739	.319–1.710	0.480				
UPS	0.901	.481–1.691	0.746				
**Tumor site** (Extremity)	0.813	0.524–1.260	0.354				
**Metastasis** (No)	3.009	1.831–4.946	**0.000**		3.702	2.089–6.562	**0.000**
**Margin status** (R0)	2.553	1.668–3.909	**0.000**		2.356	1.351–4.111	**0.003**
**Multifocal indicator** (No)	2.400	1.500–3.841	**0.000**		1.256	0.601–2.627	0.544
**Radiotherapy** (No)	0.992	0.621–1.585	0.973				
**Pharmacotherapy** (No)	1.380	0.814–2.339	0.231				
**Risk score**	1.552	1.331–1.808	**0.000**		1.450	1.184–1.776	**0.000**

HR, hazard ratio; CI, confidence interval; DLP, dedifferentiated liposarcoma; LMS, leiomyosarcoma; MFS, myxofibrosarcoma; UPS, undifferentiated pleomorphic sarcoma.

**Table 4:** Disease-free survival based on the antioxidant gene signature and clinical data for sarcoma.

	**Univariate Cox analysis**				**Multivariate Cox analysis**		
	**HR**	**95%CI**	***P***		**HR**	**95%CI**	***P***
**Age**	1.010	0.998–1.023	0.112				
**Sex** (Female)	1.086	0.765–1.543	0.644				
**Ethnicity** (Asian)							
Black	2.125	0.265–17.053	0.478				
White	1.915	0.266–13.795	0.519				
**Histological type** (DLP)							
LMS	0.799	0.514–1.243	0.320				
MFS	0.735	0.378–1.429	0.364				
Other	0.702	0.345–1.430	0.330				
UPS	0.775	0.439–1.368	0.380				
**Tumor site** (Extremity)	0.977	0.675–1.414	0.902				
**Metastasis** (No)	4.915	3.126–7.728	**0.000**		3.942	3.942–6.524	**0.000**
**Margin status** (R0)	2.085	1.422–3.056	**0.000**		1.971	1.971–3.306	**0.010**
**Multifocal indicator** (No)	2.075	1.309–3.289	**0.002**		1.493	1.493–3.077	0.277
**Radiotherapy** (No)	1.175	0.788–1.752	0.430				
**Pharmacotherapy** (No)	1.385	0.865–2.219	0.175				
**Risk score**	1.005	1.003–1.007	**0.000**		1.016	1.007–1.025	**0.000**

HR, hazard ratio; CI, confidence interval; DLP, dedifferentiated liposarcoma; LMS, leiomyosarcoma; MFS, myxofibrosarcoma; UPS, undifferentiated pleomorphic sarcoma.

**Figure 6:**
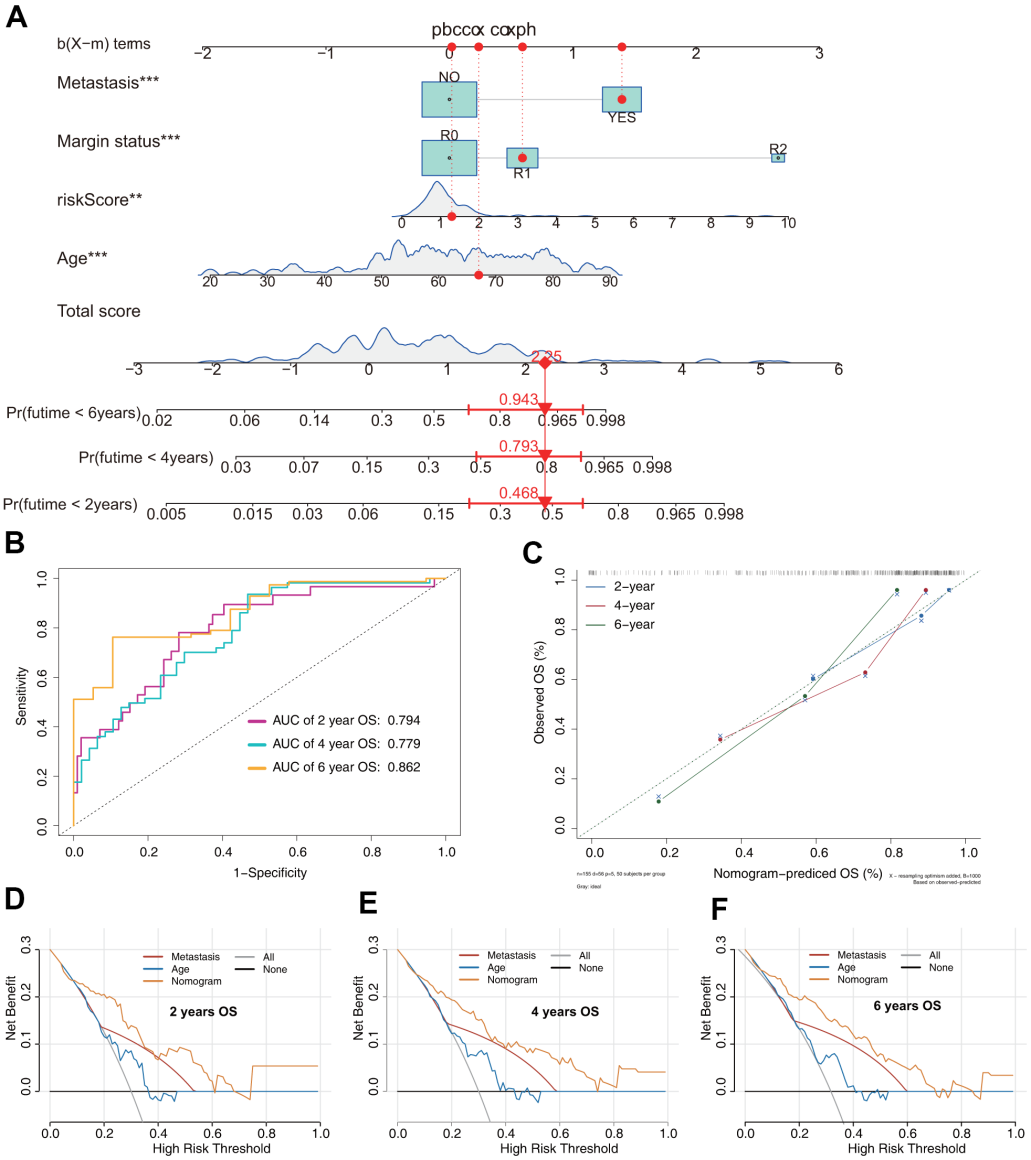
**Prediction model of OS in patients with sarcoma.** (**A**) Nomogram constructed based on the risk signature and other independent risk factors identified by Cox analysis. (**B**) ROC analysis of OS prediction in patients with sarcoma. (**C**) Calibration plot for evaluating the estimation accuracy of the nomogram. (**D**) Two-year decision curve analysis (DCA) comparing the model and other clinical features. (**E**) Four-year DCA comparing the model and other clinical features. (**F**) Six-year DCA comparing the model and other clinical features.

**Figure 7:**
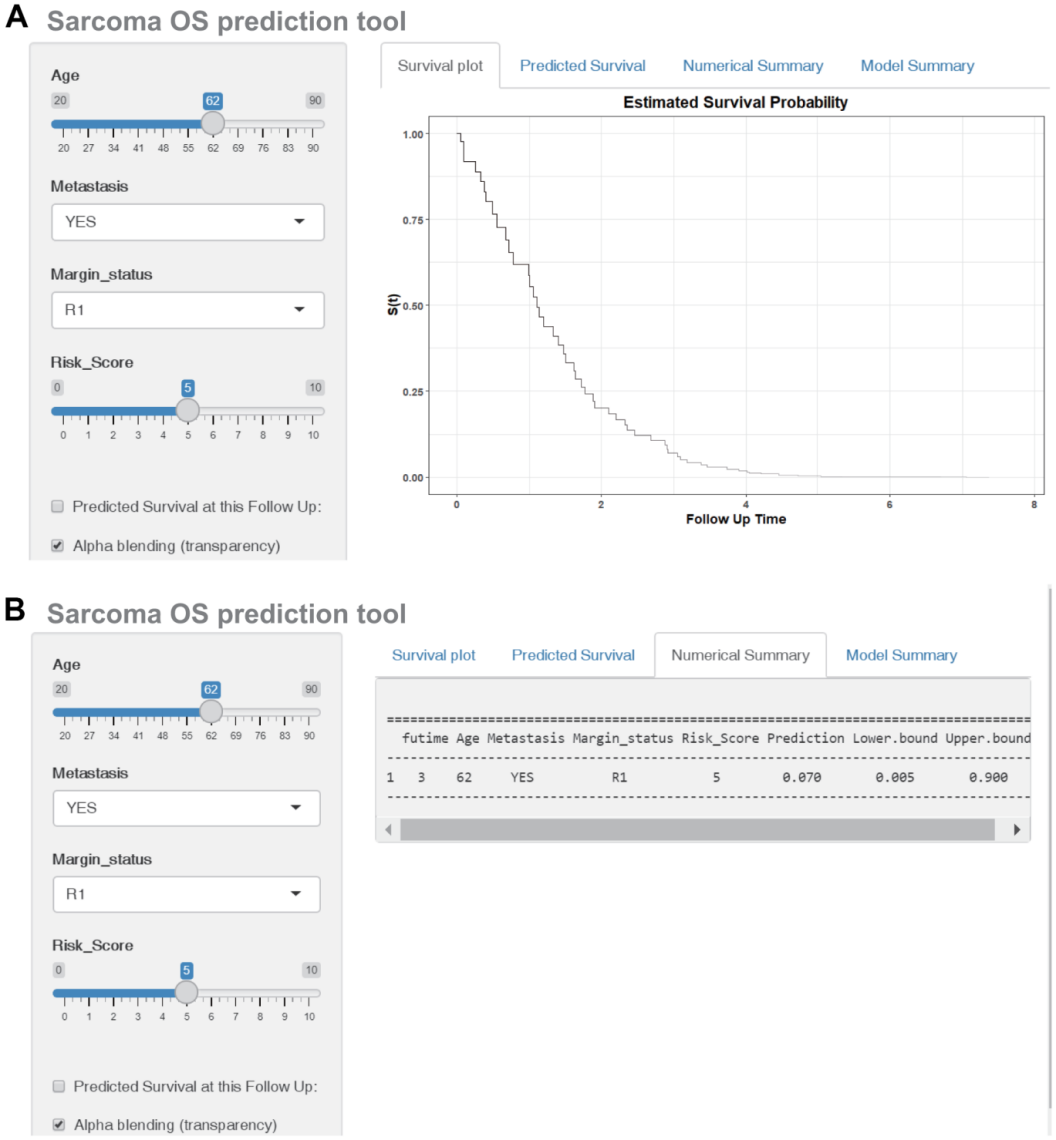
**Construction of a web-based tool (https://quankun.shinyapps.io/sarcOS/) for predicting the OS of patients with sarcoma.** (**A**) Web OS rate calculator. (**B**) Confidence interval at 95% of the web OS rate.

The OS nomogram that integrated the four independent factors is shown in Figure 6A. The predictive value of the nomogram was validated using ROC analysis, a calibration plot, and decision curve analysis (DCA). The AUCs of 2-, 4-, and 6-year OS prediction were 0.794, 0.779, and 0.862, respectively (Figure 6B). A calibration plot showed excellent calibration of the nomogram (Figure 6C). The results of DCA of the OS nomogram and other clinical features are presented in Figure 6D–6F. For the convenience of clinical application, we constructed a web-based tool (https://quankun.shinyapps.io/sarcOS/) for predicting the OS of patients with sarcoma (Figure 7A, 7B).

**Figure 8:**
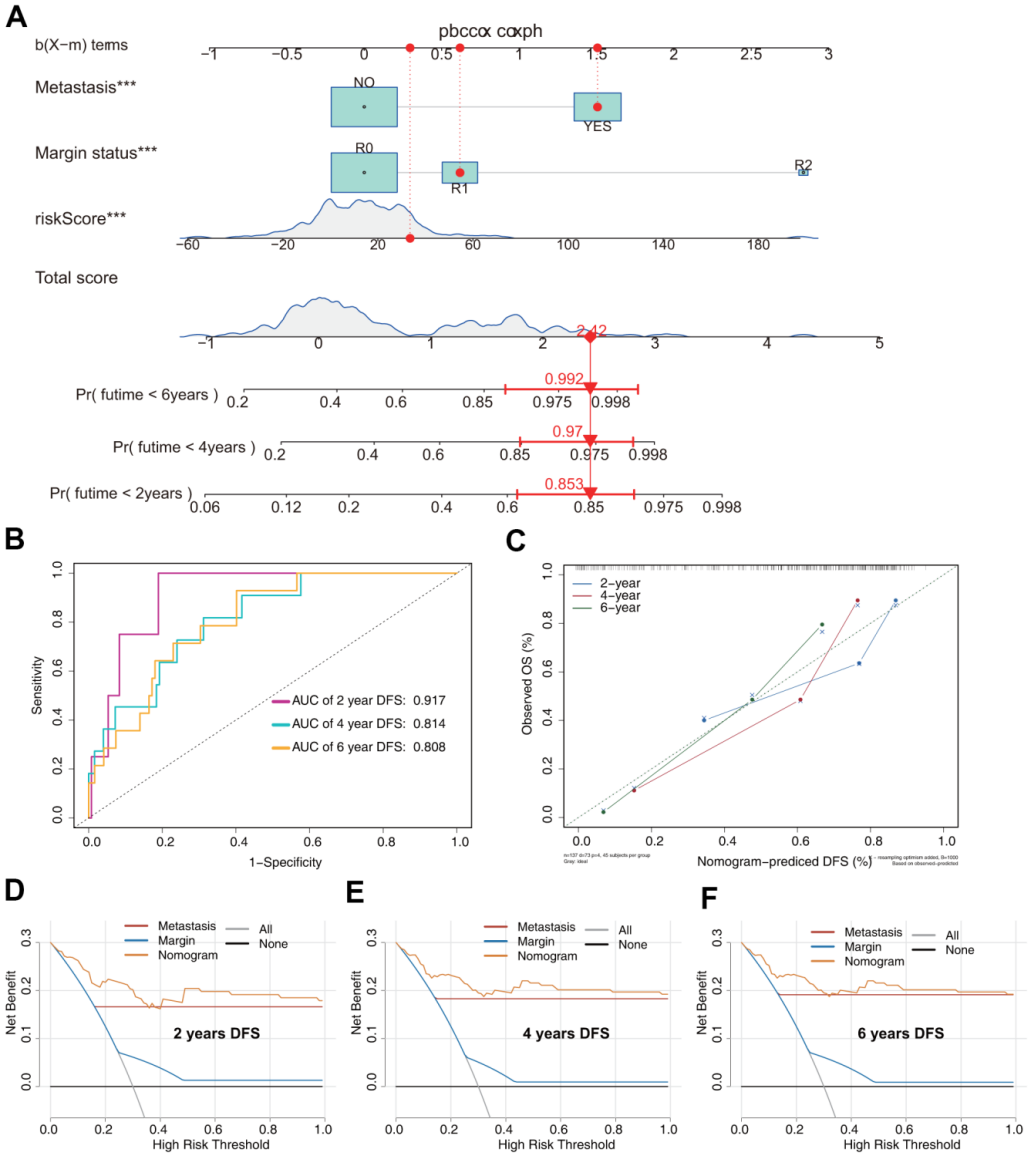
**Prediction model of DFS in patients with sarcoma.** (**A**) Nomogram constructed based on the three independent risk factors identified by Cox hazards analysis. (**B**) ROC analysis of DFS prediction in patients with sarcoma. (**C**) Calibration plot for evaluating the estimation accuracy of the nomogram. (**D**) Two-year DCA comparing the model and other clinical features. (**E**) Four-year DCA comparing the model and other clinical features. (**F**) Six-year DCA comparing the model and other clinical features.

**Figure 9:**
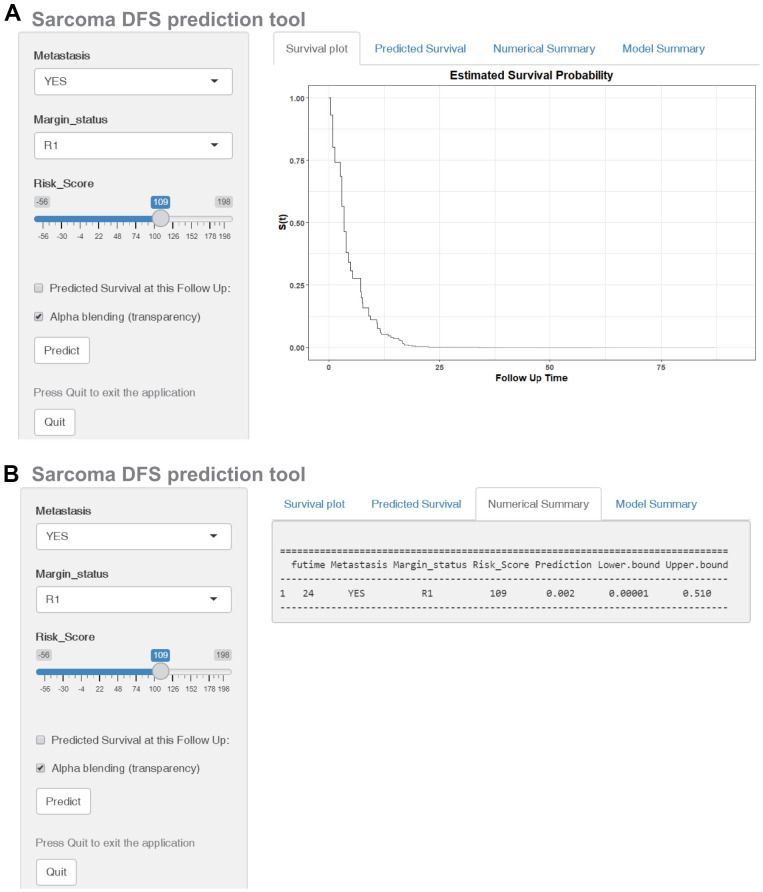
**Construction of a web-based tool (https://quankun.shinyapps.io/sarcDFS/) for predicting the DFS of patients with sarcoma.** (**A**) Web DFS rate calculator. (**B**) Confidence interval at 95% of the web DFS rate.

The DFS nomogram that integrated the three independent factors is shown in Figure 8A. ROC analysis, a calibration plot, and DCA were used to evaluate the nomogram. The AUCs of 2-, 4-, and 6-year DFS prediction were 0.917, 0.814, and 0.808, respectively (Figure 8B). A calibration plot showed excellent calibration of the nomogram (Figure 8C). The results of DCA of the DFS nomogram and other clinical features are presented in Figure 6D–6F. A web-based tool (https://quankun.shinyapps.io/sarcDFS/) was established for predicting the DFS of sarcoma patients (Figure 9A, 9B).

## DISCUSSION

Given the complex molecular regulatory mechanism of sarcoma, it is currently widely accepted that traditional tumor pathological staging cannot adequately predict the survival of patients with sarcoma [19–21]. Thus, there is a compelling need to develop novel prognostic biomarkers for sarcoma. A multitude of evidence suggests that increased ROS production plays an important role in tumor initiation and progression. The antioxidant system controls ROS production and consequently can modulate intracellular signaling pathways. Numerous antioxidant genes encode proteins involved in the antioxidant signaling pathway [22]. However, research exploring prognostic antioxidant gene signatures for sarcoma is currently lacking.

In the present study, we thoroughly investigated the implications of antioxidant genes in OS and DFS in patients with sarcoma. We systematically analyzed antioxidant gene expression profiles and survival times by LASSO regression and identified a five-gene OS signature: *CHAC2*, *GPX5*, *GSTK1*, *PXDN*, and *S100A9*. Similarly, we constructed a six-gene DFS signature: *GGTLC2*, *GLO1*, *GPX7*, *GSTK1*, *GSTM5*, and *IPCEF1*. Kaplan–Meier survival and ROC curve analyses indicated that both signatures exhibited excellent fitting and predictive ability. These results may help develop new biomarkers for the prevention and diagnosis of sarcoma and provide clinical research ideas.

Among the 10 antioxidant genes included in the OS and DFS risk signatures, few have previously been explored in terms of their association with sarcoma tumorigenesis. Liu et al. reported that *S100A9* expression was significantly increased in osteosarcoma and may be a potential marker for its diagnosis [23]. Chen et al. further investigated the underlying mechanisms and found that *S100A9* inhibited osteosarcoma cell proliferation, migration, invasion, and cell cycle by suppressing the mitogen-activated protein kinase and nuclear factor kappa B signaling pathways [24]. GLO1 expression reportedly is upregulated in several malignant tumors [25, 26]. Wang et al. explored the role of GLO1 in sarcoma development and progression and found that *GLO1* knockdown inhibited cell proliferation and migration in fibrosarcoma [27]. Although they did not evaluate the critical roles of other antioxidant genes in sarcoma development and progression, most of them are involved in malignant tumors [28–33].

To clarify the molecular mechanisms underlying the risk score, we performed GSEA and found that poor prognosis of high-risk patients with sarcoma was associated with tumor initiation, proliferation, and metastasis. In recent years, increasing attention has been paid to immune infiltration of the tumor microenvironment, which influences biological processes in the tumor. In the present study, the levels of infiltration of plasma cells, M0 and M1 macrophages, and CD8+ and CD4+ T cells were significantly different between the two risk groups. These immune cells may therefore be involved in the development of sarcoma, but this requires further investigation. A better understanding of their roles in sarcoma development may provide new prospects for immunotherapy of sarcoma.

To improve the accuracy of the prediction model, we established nomograms based on the risk scores and other independent risk factors, and these models showed good calibration and discrimination. DCA indicated that the nomograms showed higher clinical benefit and utility than simple clinical features. In addition, the AUCs for 2-, 4-, and 6-year OS prediction were 0.794, 0.779, and 0.862 for OS and 0.917, 0.814, and 0.808 for DFS, respectively. More importantly, to facilitate clinical application, we established two web-based tools to provide free services for OS and DFS prediction.

To the best of our knowledge, this study was the first to construct antioxidant gene signatures for predicting the survival of patients with sarcoma. However, the study had some limitations. First, the demographic and clinical patient data were not comprehensive. Therefore, we could not evaluate additional possible prognostic factors. The limited numbers of factors included in the models may have, at least in part, affected the precision of the nomograms. Second, only two GEO datasets were used for validation; further validation using larger datasets is needed. Third, as the prognostic roles of most of the antioxidant genes were identified for the first time in this study, *in vitro* or *in vivo* studies are needed to elucidate their specific mechanisms.

In summary, we established two antioxidant gene signatures and survival models to predict the prognosis of patients with sarcoma. These risk prediction models may serve as effective tools for designing personalized therapies and guiding medical decisions.

## MATERIALS AND METHODS

### Data collection

Transcriptomic (HTSeq-FPKM), demographic, and clinical data of patients with sarcoma were collected from the TCGA-SARC database (training cohort, https://portal.gdc.cancer.gov/) and the GEO database (validation cohorts, https://www.ncbi.nlm.nih.gov/geo/). In the TCGA-SARC dataset, after excluding cases with incomplete survival status and follow-up time, a total of 259 patients with sarcoma were included. Two GEO datasets, GSE17674 and GSE30929, comprising 44 and 140 sarcoma patients, respectively, were used to validate the OS and DFS signatures, respectively.

One hundred thirty antioxidant genes were obtained from four gene sets in the molecular signature database for GSEA (Supplementary Table 1). Data for the mutation analysis were derived from the cBioPortal for Cancer Genomics (https://www.cbioportal.org/).

### Establishment of the prognostic antioxidant gene signatures

To narrow down candidate antioxidant genes, we conducted univariate Cox regression analysis based on the TCGA-SARC dataset. OS and DFS prognostic were selected for further study. Subsequently, tenfold cross-validated LASSO regression was performed to identify potential predictors with nonzero coefficients using the R packages ‘glmnet’ and ‘survival’ [34, 35]. Finally, multivariate Cox regression analysis was performed to identify highly correlated genes and construct the OS and DFS gene signatures according to the following risk score model:


$$Risk\;score=\sum_{i=0}^{N}(\beta_{i}\times Exp_{i}),$$

where N represents the number of antioxidant genes included in the signature, Exp_i_ represents the mRNA level of the antioxidant genes included, and β_i_ represents the regression coefficient obtained by Cox regression analysis.

### Validation and assessment of the antioxidant gene signatures

To validate the antioxidant gene signatures and evaluate their prognostic value, patients with sarcoma in the TCGA-SARC and GEO datasets were classified into low- and high-risk groups according to the median value of the risk score calculated from the identified antioxidant gene signatures [36]. Survival analysis was then performed using the log-rank test to compare the difference in OS or DFS between the two risk groups. Furthermore, we investigated the time-dependent prognostic value of the signatures using time-dependent ROC curves by “survival” and “timeROC” R packages.

### GSEA

The GSEA software ((v4.0.3; http://software.broadinstitute.org/gsea/index.jsp) was used to investigate the mechanism underlying the difference in survival between the low- and high-risk groups from the training set using the KEGG gene set (c2.cp.kegg.v7.1.symbols) and GO gene set (c5.all.v7.1.symbols) [37]. For each analysis, 1,000 gene-set permutations were performed. The top five terms in each analysis were employed in multiple GSEA gene sets to demonstrate the range of biological functions and signaling pathways involved in the antioxidant gene signatures in SARC.

### Evaluation of immune cell infiltration

CIBERSORT is an analytical tool for determining the proportions of 22 immune cell types in tissues using 547 barcode gene expression values. In this study, the CIBERSORT algorithm (version 1.03; http://cibersort.stanford.edu/) was employed to determine the proportions of the 22 immune cell types in sarcoma samples [38].

### Construction of the models and web-based calculators

To obtain subsets of predictors for OS and DFS, univariate and multivariate Cox proportional hazards analyses were performed using the TCGA-SARC dataset to determine independent risk factors for nomogram construction [39]. Factors determined to be significant (*p* < 0.05) in univariate Cox regression were subjected to multivariate Cox regression analysis. Using the variables selected on the basis of the Cox regression results, we constructed combined prognostic models to evaluate the 2-, 4-, and 6-year OS and DFS of patients with sarcoma using “rms,” “Hmisc,” “lattice,” “Formula,” and “foreign” R packages. Model performance was assessed using calibration plots, time-dependent ROC analysis, and DCA. For the convenience of clinical application, web-based calculators for predicting the OS and DFS of patients with sarcoma were built using the R packages ‘DynNom’ and ‘survival’ [40].

## Supplementary Material

Supplementary Tables
