# Supplementary material for: A web-based calculator for predicting the prognosis of patients with sarcoma on the basis of antioxidant gene signatures
Source: Aging (Albany NY). 2022 Feb 10;14(3):1407–28. doi: 10.18632/aging.203885 (PMC8876918; doi:10.18632/aging.203885)
Supplement: Supplementary Tables [file aging-14-203885-s001.pdf]

SUPPLEMENTARY TABLES

Supplementary Table 1. Antioxidant genes obtained from molecular signatures database.

Gene sets	Antioxidant genes
GO_ANTIOXIDANT_ACTIVITY	ALB, ALOX5AP, APOA4, APOE, APOM, CAT, CLIC2, CYGB, DUOX1, DUOX2, EPX, FABP1, GPX1, GPX2, GPX3, GPX4, GPX5, GPX6, GPX7, GPX8, GSR, GSTA1, GSTK1, GSTM2, GSTO1, GSTO2, GSTP1, GSTT1, GSTZ1, HBA1, HBA2, HBB, HBD, HBE1, HBG1, HBG2, HBM, HBQ1, HBZ, HP, IPCEF1, IYD, KDM3B, LOXHD1, LPO, LTC4S, MGST1, MGST2, MGST3, MPO, MT3, NQO1, NXN, PARK7, PRDX1, PRDX2, PRDX3, PRDX4, PRDX5, PRDX6, PRXL2A, PRXL2B, PRXL2C, PTGS1, PTGS2, PXDN, PXDNL, S100A9, SELENOS, SELENOT, SELENOW, SESN1, SESN2, SOD1, SOD2, SOD3, SRXN1, TP53INP1, TPO, TXN, TXNDC17, TXNDC2, TXNRD1, TXNRD2, TXNRD3, UBIAD1
GO_GLUTATHIONE_METABOLIC_PROCESS	ALDH5A1, CHAC1, CHAC2, CNDP2, CTNS, DPEP1 , ETHE1, G6PD, GCLC, GCLM, GGCT, GGT1, GGT2, GGT3P, GGT5, GGT6, GGT7, GGTLC1, GGTLC2, GGTLC3, GLO1, GLRX2, GPX1, GPX4, GSR, GSS, GSTA1, GSTA2, GSTA3, GSTA4, GSTA5, GSTK1, GSTM1, GSTM2, GSTM3, GSTM4, GSTM5, GSTP1, GSTT1, GSTT2, GSTT2B, GSTZ1, HAGH, HPGDS, IDH1, MGST1, MGST2, MMACHC, NAT8, NFE2L1, NFE2L2, OPLAH, PARK7, PTGES, SLC7A11, SOD1
GO_GLUTATHIONE_CATABOLIC_PROCESS	CHAC1, CHAC2, GGT1, GGT2, GGT3P, GGT5, GGT7, GGTLC1, GGTLC2, GGTLC3
ANTIOXIDANT_ACTIVITY	APOA4, CAT, CYGB, EPX, GPX2, GPX3, GPX4, GSR, GSTZ1, IPCEF1, MGST3, MPO, PRDX2, PRDX4, SELENOS, TXNDC2, TXNRD1, TXNRD2

Supplementary Table 2A. Overall survival related genes in TCGA.

id	HR	HR.95L	HR.95H	P-value
APOM	0.694929672	0.490839127	0.983880916	0.040211656
CHAC2	1.155359794	1.019240109	1.309658286	0.02394984
CLIC2	0.967240029	0.941664334	0.993510362	0.014844272
CNDP2	0.955683908	0.915039552	0.998133611	0.040932471
GCLM	1.077595527	1.015097441	1.14394153	0.014225422
GGT1	0.836215138	0.705122233	0.991680201	0.039782991
GGT2	2977.320145	2.145235429	4132150.311	0.030256718
GLO1	1.003677441	1.002165227	1.005191937	1.83E-06
GPX5	6.95374E+11	28.73320463	1.68E+22	0.025401851
GPX7	1.008845197	1.003062881	1.014660846	0.00267565
GSS	1.031474696	1.014484921	1.048749001	0.000255122
GSTK1	0.963004499	0.940607102	0.985935214	0.00169122
GSTM5	0.868214547	0.782743445	0.963018605	0.0075259
HAGH	0.910098517	0.848010458	0.976732425	0.008975474
HP	1.046074068	1.001044359	1.093129336	0.0448072
IPCEF1	0.662818398	0.476791434	0.921426428	0.014411379
LTC4S	0.025701946	0.000705731	0.936036379	0.045933837
MGST2	0.916662086	0.867510748	0.968598235	0.00197049
NFE2L2	0.962855047	0.938390441	0.987957465	0.003943818
PRXL2C	0.923336356	0.866028848	0.98443606	0.014696048
PXDN	1.011352603	1.005330719	1.017410558	0.000211563
S100A9	0.996979959	0.994130362	0.999837724	0.038350324
SESN2	1.024347137	1.001567401	1.047644978	0.036041623
SOD2	0.987989963	0.97811544	0.997964174	0.018393386
SOD3	0.996241483	0.993136639	0.999356033	0.018057076
TXNRD3	1.249794352	1.044191608	1.495880555	0.015035818
UBIAD1	1.206410269	1.090336627	1.334840729	0.000277351

Supplementary Table 2B. Overall survival related genes in GSE17674.

id	HR	HR.95L	HR.95H	P-value
ALDH5A1	0.488242894	0.283360273	0.841265153	0.009805676
CAT	0.243388991	0.102121374	0.580076422	0.001427824
CHAC1	4.202232678	1.40897899	12.53301832	0.01002606
CHAC2	1.731501528	1.133601607	2.644754138	0.011079099
ETHE1	0.373413063	0.190002335	0.733871591	0.004269224
GGCT	3.795527295	1.511354492	9.531865303	0.004524807
GGT7	5.376836067	1.857911286	15.56068167	0.00191898
GPX5	28.36192751	1.666053041	482.8171206	0.020727186
GSTA4	0.519695482	0.280429528	0.963106117	0.037581225
GSTK1	0.234482637	0.100139376	0.549055817	0.000834435
GSTM1	0.525251397	0.281623079	0.979639279	0.042903503
GSTM2	0.499890825	0.272589871	0.916728257	0.025028079
GSTP1	3.590738658	1.200962822	10.73588947	0.022156706
GSTT1	0.448913989	0.270877539	0.743966334	0.001886978
KDM3B	0.377983896	0.174185415	0.820228412	0.013842951
LOXHD1	0.780211673	0.633213122	0.961335502	0.019796816
MT3	3.115568068	1.028136039	9.441128426	0.044535257
PRDX4	2.206151533	1.111739092	4.377919804	0.023641774
PTGES	51.64172666	8.342992391	319.6536456	2.23E-05
PXDN	1.913703301	1.133051434	3.232210132	0.015220892
S100A9	1.50226258	1.024294983	2.203264582	0.037268071
SESN1	0.511400709	0.295158992	0.886067144	0.016788577
SRXN1	2.483881638	1.065109271	5.792521163	0.035207005
TP53INP1	0.0642593	0.012640652	0.326664912	0.000937732
TXN	2.906414261	1.353150059	6.242651213	0.006231613
TXNRD1	8.611899948	1.635886184	45.33617401	0.011061466

Supplementary Table 3A. Disease-free survival related genes in TCGA.

id	HR	HR.95L	HR.95H	pvalue
CHAC2	1.138053912	1.018636202	1.27147131	0.022229274
DPEP1	1.072903191	1.018251369	1.130488298	0.008339277
GCLM	1.074256007	1.020313647	1.131050215	0.006429505
GGT6	2.576896399	1.248951694	5.316774929	0.010420976
GGTLC2	3.52E-08	7.28E-16	1.701346374	0.057289229
GLO1	1.002707912	1.001189807	1.004228319	0.00046849
GPX2	1.042398062	1.01771136	1.06768359	0.000684666
GPX7	1.008581171	1.002829263	1.01436607	0.003409695
GSS	1.026499459	1.008810868	1.044498204	0.003186986
GSTA1	1.009963512	1.00025664	1.019764583	0.044215604
GSTK1	0.981308861	0.963305916	0.999648258	0.045803157
GSTM3	0.967616257	0.938544384	0.997588645	0.034423142
GSTM5	0.946494025	0.895208412	1.00071774	0.053024547
IPCEF1	0.815225628	0.661366383	1.004878447	0.055574138
IYD	212.9982566	4.328479785	10481.33746	0.006995491
NFE2L2	0.979518998	0.958478803	1.00102106	0.061783231
PXDN	1.008177734	1.001928685	1.014465758	0.010247922
UBIAD1	1.100932343	0.997005815	1.21569203	0.057342526

Supplementary Table 3B. Disease-free survival related genes in GSE30929.

id	HR	HR.95L	HR.95H	pvalue
ALDH5A1	0.422656461	0.254279875	0.702527023	0.000894227
APOA4	0.166787005	0.029183725	0.953199249	0.044024404
CAT	0.361442798	0.221818748	0.588953354	4.40E-05
DUOX1	0.243716631	0.070936925	0.837332553	0.024968334
FABP1	0.00711812	0.000367002	0.138058273	0.001079862
GCLC	0.595187031	0.364860393	0.970912735	0.037691793
GGCT	3.186203687	1.773105142	5.725488971	0.000106523
GGTLC2	0.170838953	0.037589682	0.776435084	0.022164081
GLO1	3.607525959	1.470502598	8.850200988	0.00507672
GPX1	0.597193306	0.376224957	0.94794308	0.02876173
GPX3	0.794340534	0.663901276	0.950407697	0.011878723
GPX4	0.583327575	0.399984116	0.850711432	0.00511338
GPX5	0.036287858	0.002362546	0.55736834	0.017343185
GPX7	1.916849565	1.359856538	2.701985213	0.00020334
GSTK1	0.294243084	0.173421357	0.499240657	5.75E-06
GSTM1	0.453597006	0.267535106	0.769058863	0.003337836
GSTM2	0.50868778	0.326994953	0.791337159	0.002717673
GSTM5	0.494121613	0.29746479	0.820790145	0.006475419
GSTT1	0.647055994	0.483738929	0.865511196	0.003355428
GSTT2	2.235335435	1.195607831	4.179233672	0.011750317
GSTZ1	0.44505875	0.227745663	0.869730243	0.017871424
HAGH	0.2985334	0.120372143	0.74038884	0.009092119
HBB	0.695501245	0.57076775	0.847493542	0.000317194
HBD	0.101332154	0.030971362	0.331538719	0.000153432
HBE1	0.060803564	0.00493651	0.748924583	0.028842283
IDH1	0.58100736	0.410866954	0.821603074	0.002130104
IPCEF1	0.262314203	0.073360619	0.937952028	0.0395423
MGST2	0.310465095	0.141445585	0.681453404	0.003543972
MGST3	0.657811909	0.475411373	0.910193849	0.01147497
NQO1	0.559752863	0.379814771	0.824937027	0.00336159
NXN	2.196883781	1.533874622	3.146475128	1.76E-05
PRDX1	2.10332709	1.044610909	4.235055185	0.037325306
PRDX3	0.13658556	0.038566147	0.483730333	0.002031811
PRDX4	1.50849613	1.005299292	2.26356528	0.047090029
PTGS1	1.759009538	1.177358155	2.628014714	0.005832676
SLC7A11	4.915350344	2.147831408	11.24886661	0.000163426
SOD3	0.459144337	0.305881816	0.689199266	0.000172551
UBIAD1	3.586817718	1.247755998	10.3107189	0.017748622
